# Perioperative oliguria: adequate physiological response or risk for acute kidney injury?

**DOI:** 10.1590/2175-8239-JBN-2020-E001

**Published:** 2021-02-10

**Authors:** Renata de Souza Mendes, José Suassuna

**Affiliations:** 1 Universidade do Estado do Rio de Janeiro, Hospital Universitário Pedro Ernesto, Rio de Janeiro, RJ, Brasil.; 2 Universidade Federal do Rio de janeiro, Hospital Universitário Clementino Fraga Filho, RJ, Brasil.

Acute kidney injury (AKI) is a severe complication in the surgical setting, being associated with increased costs^1^ and adverse clinical outcomes, both in the short and in the long term^2^^,^ 3. In addition, AKI's multifaceted etiology and complex pathophysiology may hinder the adoption of universal preventive measures^4^. The identification of specific risk factors for perioperative AKI is essential for the development of appropriate prevention and treatment strategies. Some factors, such as age, race, obesity, and pre-existing diseases, are not modifiable. Others, such as prolonged surgical time, manipulation of large vessels, hemodynamic instability, and exposure to nephrotoxic drugs, are dependent on medical performance^5^.

At present, the diagnosis of AKI is based on two functional criteria: serum creatinine and urine output (UO). Although they are the mainstay of the current AKI severity stratification systems, such as RIFLE, AKIN, and KDIGO, both lack precision in the perioperative period^4^. The increase in serum creatinine is a late response and tend to be minimized by volume replacement and by its decreased production after major surgery or critical illness. Urine output, on the other hand, lacks reliability, has no etiological specificity, is affected by the use of diuretics during the anesthetic time, and first and foremost is influenced by physiological responses.

Almost all major surgeries are performed under a physiological antidiuretic state. This is due to the necessary due to the necessary pre-anesthetic fasting, which is aggravated by the use of narcotic and anesthetic agents and by the manipulation of viscera and peritoneum^6^. There is also reflex vasoconstriction, mediated by neuroendocrine response, surgical trauma, blood loss, insensitive fluid losses, and mechanical ventilation.

Additional hemodynamic influences, which may include vasodilation and myocardial depression, are caused by anesthesia, especially when volatile agents are used. Thus, to maintain the kidney and other organs' perfusion, the anesthetic management requires the replacement of crystalloid or colloid solutions and, occasionally, the use of vasoconstrictors. In this delicate balance, the intrinsic self-regulation of renal blood flow and glomerular filtration rate are involved, as well as the sympathetic and the renin-angiotensin-aldosterone systems, and the antidiuretic hormone axis. It is not uncommon that the fluid balance restoration results in a transient physiological reduction in UO. Another relevant point is the association of positive perioperative fluid balance with the risk of complications^7^, which has led to a trend of more restrictive strategies for volume replacement during anesthesia and lower perioperative urine production.

It appears, therefore, that a decrease in urine output as a criterion for AKI in the perioperative scenario is not specific, as it lacks association with unfavorable outcomes, including the development of AKI, length of hospital stay, and mortality^8^^,^ 9. This, however, is not a universal observation^10^^,^ 11.

This BJN issue presents the work "Intraoperative oliguria does not predict postoperative acute kidney injury in major abdominal surgery: a cohort analysis"^12^, by Inácio and collaborators. In a series of 165 patients undergoing major elective abdominal surgery, the authors defined perioperative oliguria as urine output of less than 0.5 mL/kg/h, which occurred in only 20 patients. The small number of participants and, mainly, of the cases with the outcome of interest, compromised the statistical power of the study. Even so, once again, no association was found between intraoperative oliguria and the development of KDIGO-defined AKI, and no impact on hospitalization time and mortality was observed.

The concluding message is that, perhaps, the "urine output" criterion of the current AKI's stratification systems is not applicable in the operating room. In a proportion of patients, perioperative oliguria might be just the expression of the physiological response to trauma, exposure to anesthetic agents, and the loss of fluid that occurs before and during the procedure.

In addition to the risk involved in excessive volume resuscitation, the use of perioperative oliguria as an isolated perfusion marker should be avoided, given its limited impact.

In the current scenario, where increasingly complex surgical procedures are performed on increasingly frail patients, knowledge about the renal response to surgery and anesthesia, the adequacy of volume replacement, preferably guided by dynamic parameters^13^, and attentive care during and after the surgical stress are far more powerful weapons than any single parameter to minimize the risk of post-operative AKI.
